# Defining digital coaching: a qualitative inductive approach

**DOI:** 10.3389/fpsyg.2023.1148243

**Published:** 2023-10-05

**Authors:** Sandra J. Diller, Jonathan Passmore

**Affiliations:** ^1^Management Faculty, Private University Seeburg, Seekirchen am Wallersee, Austria; ^2^LMU Center for Leadership and People Management, Social Psychology Division, Ludwig Maximilian University of Munich, Munich, Bavaria, Germany; ^3^Institute of Coaching, McLean and Affiliate of Harvard Medical School, Boston, MA, United States; ^4^Henley Business School, University of Reading, Reading, United Kingdom

**Keywords:** digital coaching, distance coaching, e-coaching, online coaching, remote coaching, AI coaching

## Abstract

The term ‘digital coaching’ is widely used but ill-defined. The present study therefore investigates how digital coaching is defined and how it differentiates from face-to-face coaching and other digital-technology-enabled (DT-enabled) formats, such as digital training, digital mentoring, or digital consulting. A qualitative inductive approach was chosen for more in-depth and open-minded content. Based on previous studies on the importance of asking coaches working in the field, 260 coaches working in the field of digital coaching were surveyed. The given answers depict the importance of differing between forms of DT-enabled coaching. Thus, *digital coaching* is a DT-enabled, synchronous conversation between a human coach and a human coachee, which is different to *artificial intelligence* (AI) coaching and coaching that is supported by asynchronous digital and learning communication technologies. Due to this definition and differentiation, future studies can explore the digital coaching process and its effectiveness – particularly in comparison to other formats. Furthermore, this clear definition enables practitioners to maintain professional standards and manage client’s expectations of digital coaching while helping clients understand what to expect from digital coaching.

## Introduction

Coaching in the organizational context has emerged over the past two decades as not only a popular but also effective human resource development intervention that can have beneficial outcomes on an individual and organizational level (Grover and Furnham, 2016; International Coaching Federation, 2021; DeHaan and Nilsson, 2023; Passmore et al., 2023). Previous research has thereby defined coaching and differentiated it from other formats like training or consulting by viewing it as a synchronous intervention *to empower clients to attain their self-valued goals in their self-determined way* by the use of conversation management techniques like open questions or active listening, which are aimed at stimulating the self-awareness and self-determination of the client (Grant et al., 2010; Passmore and Fillery-Travis, 2011; Greif et al., 2018; Passmore and Lai, 2019; Diller et al., 2020). Due to the development of digital communication tools and due to the COVID-19 pandemic, coaching was increasingly delivered in a digital environment (International Coaching Federation, 2021; Passmore, 2021). Yet, digital coaching has so far not been the subject of in-depth review with “an apparent lack of consensus around its meaning” (Geissler et al., 2014, p. 166). In addition to digital coaching being ill-defined, there have been many names for this new digital method of coaching, including e-coaching, virtual coaching, distance coaching, online coaching, and remote coaching (e.g., Berry et al., 2011; Rock et al., 2011; Ribbers and Waringa, 2015; Jackson and Bourne, 2020; Crawford et al., 2021). This missing definition leads to a lack of clarity about the boundary: When should the intervention be called ‘digital training’ or ‘digital mentoring,’ and when ‘digital coaching’? This issue of boundary can be found in recent publications that call an artificial intelligence (AI) training intervention “digital coaching” (e.g., Allemand et al., 2020; Allemand and Flückiger, 2022) or refer to “digital coaching” when using a training watch that measures and adjusts the person’s physiological information, personal fitness goals, and achievements (Kettunen et al., 2022). With coaching being an unregulated profession with anything being called ‘coaching’ (Greif et al., 2018; Kühl, 2021), such issues easily emerge, highlighting the importance of a clear definition towards a professionalization of coaching. This lack of a shared definition of digital coaching not only complicates theory development but also empirical exploration: “Without an agreed upon explicit definition which outlines underlying assumptions and boundaries of the concept, it is challenging, if not impossible, for the literature to develop further. Clear conceptualization is required to ensure that attention can be turned to the development and subsequent testing of a theory [...]. Such a conceptualization is also essential for enabling organizations to understand what exactly they are purchasing and why” (Jones et al., 2019, p. 62). Thus, the present research contributes to the definition of digital coaching with a data-driven qualitative inductive approach by questioning practitioners in the field.

### The advantages and risks of digital technologies in coaching

With the emergence of digital communication tools, not only digital business communication but also digital learning, development, and support became more prominent, even though the majority was still taking place face-to-face before the COVID-19 pandemic (Sugrue and Rivera, 2005; Cowling, 2016; König et al., 2017; Passmore et al., 2021). This changed significantly with the COVID-19 pandemic with over half learning taking place in virtual learning environments (Ken Blanchard Companies, 2021). The advantage of technology is to enable on-demand learning, development, and support with people from around the world (Sugrue and Rivera, 2005; Taylor et al., 2008; Radu et al., 2011; Haleem et al., 2022). Furthermore, AI as virtual interaction partners can enable people to open up about personal information due to reducing impression management and fear of negative evaluation (Suler, 2004; Gratch, 2014). Accordingly, digital learning environments are used in several organizations as they are perceived as low in cost but high in impact (Ensher et al., 2003; Bierema and Hill, 2005; Sousa and Rocha, 2019). Organizations use social networking software, centralized electronic knowledge-sharing systems, and Web 2.0 technologies in teaching and learning (Kulakli and Mahony, 2014). Thus, digital technologies enable new opportunities for individual support and behavior change, as it can be independent of space and sometimes even independently of time (Nahum-Shani et al., 2018). Even more so, digital technologies can help reduce time and travel expenses of attending a session, as well as help people that would not be able to travel (e.g., disabilities, or reduced travel options) (e.g., long-distance) (Amichai-Hamburger et al., 2014). In addition, digital technologies support more flexible and open communication due to options for when, how (often), and between whom communication takes place (Hamilton and Scandura, 2003; Wainfan and Davis, 2004).

The use of digital technology in the coaching context is part of this shift to more just-in-time learning and performance support (Brandenburg and Ellinger, 2003; Kim et al., 2005; Hernez-Broome et al., 2007; Passmore and Evans-Krimme, 2021). Similar to the use of digital technology for learning and development, it provides “a variety of means for synchronous and asynchronous communication [which can] alter the timing, scheduling, and formality of the coaching process” (Frazee, 2008, p. 7), leading to cost-effective and easily accessible coaching solutions (Barbian, 2002; Charbonneau, 2002; Sparrow, 2006). Secondly, digital technologies in coaching have the potential of monitoring thoughts, feelings, behavior, or processes, which can be useful for clients (e.g., reflecting their feelings or behavior in a situation from an outside view) and coaches (e.g., coach supervision) (Rossett and Marino, 2005; Tausczik and Pennebaker, 2010; Amichai-Hamburger et al., 2014; Marsch et al., 2014; Trull and Ebner-Priemer, 2014; Allemand et al., 2020; Harari et al., 2020). Thirdly, digital environments are sometimes perceived as safer and more secure spaces than face-to-face environments by users (Hamburger and Ben-Artzi, 2000), which can help to talk openly about issues (Miyahira et al., 2012). Fourthly, digital environments can enhance the self-development process with new self-change opportunities, such as using a certain avatar to make yourself feel stronger (proteus effect; Yee and Bailenson, 2007), to explore roles and identities (Slater et al., 2010), or to reflect on body images (virtual embodiment; Hänsell et al., 2011; Normand et al., 2011; Riva, 2011).

Research in the psychotherapy field indicates that digital interventions could be as effective as face-to-face interventions (e.g., Day and Schneider, 2002; Andrews et al., 2018; Weightman, 2020). Similarly, coaching-related training apps showed positive outcomes on short-term personality change and self-control (e.g., Schueller et al., 2013; Nahum-Shani et al., 2018; Allemand et al., 2020; Stieger et al., 2021; Allemand and Flückiger, 2022; Kettunen et al., 2022). Furthermore, first studies on phone/chat coaching indicate similar results, showing beneficial effect on goal clarification, goal attainment, subjective well-being, the coach-client relationship and coaching satisfaction (Ghods, 2009; Berry et al., 2011; Poepsel, 2011; Kim and Lee, 2023; Wang et al., 2023) - while noting that these studies have their limitations based on their experimental design, sample size, and the specificity of coaching offer. Likewise, coaching-related video consulting approaches seem to not conflict with the coach-client relationship and the coaching effectiveness (Carson and Choppin, 2021; Bak et al., 2023). In addition, two recently published studies comparing digital, face-to-face, and blended coaching have shown similar effectiveness in terms of coaching success as percived by clients and coaches (Doyle and Bradley, 2023; Michalik and Schermuly, 2023). Moreover, AI coaching positively affected goal attainment with no differences compared to a human coach (Terblanche et al., 2022). In sum, digital coaching can be an effective coaching approach.

Yet, not every coaching approach is more effective online (Bak et al., 2023; Kim and Lee, 2023) and there are risks when using digital tools in coaching. Firstly, interventions in a digital environment can reduce the visibility of nonverbal cues, potentially impacting the development of trust and adversely impacting the working alliance, leading to less openness, commitment, and goal attainment (Wells et al., 2007; Scharff, 2013; Amichai-Hamburger et al., 2014; Feijt et al., 2020). For instance, coaches found active listening and the interpretation of pauses in a digital coaching environment difficult (Frazee, 2008). This reduced visibility of non-verbal cues is particularly concerning for critical situations (Charbonneau, 2002; Amichai-Hamburger et al., 2014). Consequently, one is forced to concentrate intensively on the course of the conversation in order not to miss any information (Bailenson, 2021), which can much more easily lead to increased exhaustion (‘Zoom fatigue’; Shockley et al., 2021). For example, mental health practitioners found sessions *via* video communications tools more draining and tiring than face-to-face sessions (Feijt et al., 2020). A second risk in the digital setting are disruptions in the physical space (Meyer, 2023) but also in the digital room, such as *via* delays in video or voice, technical faults with the software or connection, failures in power supply, incoming emails notifications, or automated software updates (Amichai-Hamburger et al., 2014). A recently published study comparing digital and face-to-face coaching has shown that more side effects were perceived by coaches and clients in the digital environment (Michalik and Schermuly, 2023).

In sum, using digital technologies for learning and development can have several benefits concerning costs, time, and space, and therefore availability. Furthermore, it could be that digital coaching shows a similar effectiveness in terms of goal striving, relationship building, and well-being. Yet, risks may occur that include the decrease of nonverbal cues and the possibility of technical difficulties. To better research the effectiveness of digital coaching, digital coaching itself needs to be defined first.

### The present research: defining digital coaching

Coaching conducted through digital means has been referred to by various terms in the literature, such as e-coaching, i.e., “a non-hierarchical developmental partnership between two parties separated by a geographical distance, in which the learning and reflection process is conducted *via* both analogue and virtual means” (Ribbers and Waringa, 2015, p. 6), virtual coaching, i.e., “a coach interact[ing] electronically” (Rock et al., 2011, p. 42), distance coaching. i.e., “any interaction between the coach and the client that is not face-to-face” (Berry et al., 2011, p. 244), online coaching, i.e., *via* “electronic devices, such as a computer, laptop, tablet or smart phone with an internet connection, without the need for travel [where coach and client do not] come together physically” (Jackson and Bourne, 2020, p. 21), or remote coaching, i.e., “coaching delivered through technology” (Crawford et al., 2021, p. 1610). This list highlights the problem of a clear terminology, creating a challenge for establishing a clear definition in the field. In addition to the lack of consensus on a term, there is ambiguity regarding the boundaries between digital coaching and other formats. These missing boundaries lead to an emerging research on so-called “digital coaching” when testing digital training apps without a human at the coach’s end (Allemand et al., 2020; Allemand and Flückiger, 2022; Kettunen et al., 2022; Hopman et al., 2023; Santini et al., 2023), describing digital coaching as “any computer program that supports spoken, text-based or multimodal conversational interactions with humans, such as personal digital assistants, virtual personal assistants, conversational agents or chats” (Santini et al., 2023). Thus, a clear definition and differentiation of digital coaching is needed to prevent an even more unregulated use of the term coaching, to develop the digital coaching literature further, and to enable the practitioner’s understanding on what exactly they are purchasing and why.

The present research contributes to the definition of coaching *via* a data-driven qualitative inductive approach to differentiate and define a coaching term. Such a qualitative approach is suitable when something is underspecified theoretically and helps to “capture and describe depth, richness, and complexity of phenomena” (Arino et al., 2016, p. 109). In this regard, the viewpoint of practitioners is of particular importance when a phenomenon is theoretically underspecified and also more practitioner- than theory-led (Shepherd and Suddaby, 2017; Jones et al., 2019). This practitioner-oriented approach enables to “derive and shape the development of an inclusive definition” (Jones et al., 2019, p. 63). By differentiating digital coaching from similar interventions, the present research further explores specific differences between digital coaching and face-to-face coaching as well as other digital interventions. These two key contributions of defining and differentiating digital coaching help to shift the research agenda from understanding digital coaching to exploring the process and its effectiveness.

## Method

### Sample

The online survey was shared *via* social media channels of private coaches, professional coaching bodies, and coaching networks between January and April 2022. In total, 260 participants (168 women, 88 men, one non-binary/third gender, one genderqueer, and two agender) between 23 and 77 years old (*M* = 51.05, *SD* = 9.08) completed our web-based survey. Coaches participated from over 40 countries around the world with most coaches coming from the United Kingdom (*n* = 137); other countries had between one to twelve participating coaches. Most coaches were external coaches (*n* = 193) (internal: *n* = 24; both: *n* = 43) with the whole variance of never having experienced supervision to very often using supervision (*M* = 3.37, *SD* = 1.10; scale ranging from 1 to 5). The surveyed coaches had 565.30 h on average of coach training background (*SD* = 1606.22) as well as 9.28 years on average of coaching experience (*SD* = 7.28).

With regard to their experience in digital coaching, only coaches with digital coaching experience and who have had more than zero coaching sessions were assessed. A bit more than half (60%) of the coaches indicated to have started using digital coaching during the COVID-19 pandemic while the others have already used it before the pandemic. On average, the surveyed coaches now offer mainly digital coaching: 88% of their coaching are digital (*SD* = 22%) and 11% face-to-face (*SD* = 22%) (1% *via* other formats (unspecified); *SD* = 1%).

### Design and measures

The online survey started with an introduction to the study and an informed consent that the participants had to actively agree to in order to proceed with the survey. Then, demographical questions and questions on their coaching background as well as digital coaching background were asked. Here, questions also included “How long does a regular *digital* session last (in minutes, i.e., 30, 60, 120 min,...)?” and “How long does a regular *face-to-face* session last (in minutes, i.e., 30, 60, 120 min,...)?”.^1^ At the end, the participants were asked three open-ended questions based on the design of Jones et al. (2019; original questions regarding team coaching: “How do you define team coaching?”, How is team coaching different to one-to-one coaching?” and “How is team coaching different to other team development interventions?”): “How do you define *‘digital coaching’*?”, “How is *digital coaching* different from face-to-face coaching?”, and “How is *digital coaching* different from other digital development interventions (e.g., digital training, digital mentoring, digital consulting)?” The three questions were intentionally designed to be flexible and open-ended. The survey was piloted with a small sample (*n* = 5), checking face and content validity.

### Data analysis

The data were analyzed with an inductive approach, which is the appropriate method when there are no pre-existing categories (Flick, 1992; Mayring and Fenzl, 2017), using Mayring’s (2001, 2012) seven-step approach for qualitative content analysis (see also Schiemann et al., 2019, with other coaching data using the same approach). Both research-independent inter-coders (a man and a woman; both with a psychology and qualitative content analysis background) coded all responses independently of each other with a low to middle level of abstraction and no double-coding allowed. To ensure reliability, the passages were only analyzed when both inter-coders agreed (100% intercoder agreement). Due to the inductive approach, there was no minimum or maximum for subcategories per question. Both coders discussed and agreed on the category names together. For this analysis, the QCAmap software was used^2^ (Mayring and Fenzl, 2017). Quantitative data, including the demographic data, the number of minutes per average session and the within subject t-test, were analyzed using SPSS 24.0. The dataset is openly available on Open Science Framework.^3^

## Results

### How digital coaching is defined

Based on this open question, most coaches referred to digital coaching as coaching with video/visual/seeing each other (*n* = 154) or in virtual rooms (*n* = 28) with less describing it to be *via* audio/telephone (*n* = 48): As one coach described, digital coaching is “face-to-face coaching by [a] camera using digital means, typically [a] laptop, pc or smartphone” (#119). To give another example, #150 described digital coaching as “the same as face-to-face coaching except we use a digital format, e.g., Teams, Zoom or Skype to support a client to gain awareness and clarity of their skills, strengths, and resources, with a view to moving towards a goal that may be related to performance, wellbeing or some other area of their life.” Thus, digital coaching was mostly described as a synchronous coaching technique; some coaches even underlined the importance of synchronicity (*n* = 12). Digital coaching was further described to be technology-based (*n* = 151) / online (*n* = 112) / remote (*n* = 13). Another aspect named was the interaction with a human coach (*n* = 27): “Digital for me encompasses all things virtual but includes a real person (i.e., coach)” (#197). Some coaches further described possibilities of add-on support of the client *via* asynchronous technology such as *via* email, chat, an app, or a support platform (*n* = 37). AI was mentioned by a few as a possible substitute for the human coach (*n* = 4), for matching coach and client (#65), and using it as an additional tool (#32). Some coaches further pointed out the independence of time or place (*n* = 36) as well as high accessibility (*n* = 13) in their definitions.

### How digital coaching differs from face-to-face coaching

A first difference can be seen with regard to the session duration: The coaches’ face-to-face coaching sessions were with an average of 76.33 min (*SD* = 22.66) significantly longer than their digital coaching sessions (*M* = 63.86, *SD* = 18.06), within-subject *t*(239) = 10.09, *p* < 0.001. Further differences were found in the second open question: While 32 coaches reported no concrete differences, the other coaches listed evaluative differences with regard to advantages and disadvantages concerning for instance the accessibility, flexibility, communication, and relationship. As one coach said, “both offer advantages and disadvantages, therefore, rendering digital and face-to-face coaching more effective in certain situations” (#330). Coaches described digital coaching compared to face-to-face coaching as an easier procedure (e.g., flexibility, no travel, preparation, no room renting, more convenient, higher efficacy) (*n* = 75) with therefore greater accessibility (*n* = 34) and sustainability (*n* = 2). They further see an advantage in the use of technology as support to share information (*n* = 16) and the feeling of comfort / safety at home (*n* = 21). However, digital coaching compared to face-to-face coaching has a loss of nonverbal communication (e.g., less non-verbal cues, no feeling of the atmosphere) (*n* = 91) with the coach needing to pay more attention / listen more deeply (*n* = 17) or the coaching not being as impactful as face-to-face (*n* = 3). Furthermore, it is more difficult to build up a relationship (e.g., less personal/intimacy, longer time to get to know each other, no physical presence, no connecting besides the coaching) (*n* = 53). In addition, there can be technological disadvantages (e.g., disruptions, distraction, limitations of tools) (*n* = 28) and additional requirements for the coach (*n* = 16).

### How digital coaching differs from other digital development formats

Coaches described digital coaching in comparison to other digital formats as having the same differences as face-to-face coaching from other face-to-face development formats, such as training or mentoring (*n* = 121). Other coaches underlined the more personal approach in (digital) coaching compared to other formats (*n* = 36) with the client being much more empowered to self-learn, self-reflect, and self-change (*n* = 32). Others described digital coaching as more challenging than other digital formats, as it is more complex (*n* = 23), and as it needs more engagement (*n* = 15). In addition, digital coaching is expected to need fewer tools (*n* = 16) and is perceived as a safer environment (*n* = 3) than other formats. A few coaches see these formats as similar, as being similarly (non) effective (*n* = 3) or missing the vital social connection compared to face-to-face formats (*n* = 8).

## Discussion

Given the rapid emergence of global digital coaching providers, such as BetterUp, CoachHub, or EZRA, as well as the widespread use of digital coaching, coaching science needs to catch up. Billions of dollars have already been invested in these new businesses by venture capital, and the next 5 years, 2023–2028 are likely to see one or more of these companies come to the market as IPOs with a multi-billion valuation (Bersin, 2022). However, a shared language for researchers, practitioners, and investors is needed on what exactly digital coaching is. The present research, therefore, explored the definition and differentiation of digital coaching compared to face-to-face coaching and other digital development formats. With a clear definition, this paper can provide a first step in building both theory and research in this new field. Based on a first finding, digital coaching is described as a human-to-human interaction. This differentiates digital coaching from AI/non-human coaching (human-to-AI/computer interaction) (Grassmann and Schermuly, 2021). A second finding is that digital coaching is seen as a synchronous support, which differentiates it from the use of additional asynchronous digital communication technologies (e.g., text messaging, email, apps, or learning platforms) in digital or face-to-face coaching. Thirdly, digital coaching was described as very similar to face-to-face coaching in its process – particularly in comparison to other digital formats. Thus, ‘digital coaching’ can be defined as a *synchronous, personal conversation using DT-enabled audio and/or video channels of communication between a human coach and a human coachee to empower the coachee in their self-development*.

In addition to its similarities to a face-to-face coaching approach, we further found coaches *perceived* (dis)advantages for using the same approach in an offline versus online environment. Thus, the approach in the digital environment has advantages of accessibility, flexibility, and feeling of safety at home while it has disadvantages of zoom fatigue, loss of nonverbal communication, and challenges in relationship-building. These (dis)advantages of the digital environment are in line with other very individual interventions (e.g., Amichai-Hamburger et al., 2014). In addition, a digital coaching sessions was reported to be shorter than a face-to-face session. This difference could underline the reported exhaustion in a digital environment due to more attention to nonverbal cues and nonverbal overload (Bailenson, 2021). This challenge was also named by differing digital coaching from other digital interventions, requiring greater engagement due to its complexity. In addition, video meetings can cause zoom fatigue due to a negative attention towards oneself and enhanced self-evaluation, making you aware of your own presence in a negative way (Wiederhold, 2020; Bailenson, 2021; Shockley et al., 2021; Ratan et al., 2022). Thus, the attention shifts from the client and the process towards the coach, which could affect coach presence (Abravanel and Gavin, 2021). A further named disadvantage concerned the relationship and communication itself. While the working alliance might not have been affected (Berry, 2005; Berry et al., 2011), the coaching relationship could go beyond the working alliance (Diller et al., 2022). In particular, empathy and trust as essential coaching success factors might be more difficult in a digital environment, when being shown nonverbally (Schiemann et al., 2019; Diller et al., 2021, 2023). In sum, more research is needed to explore the different processes for face-to-face compared to digital coaching among both coach and client.

## Limitations

We recognize that our study has some limitations. The first limitation concerns the group of participants, as we have concentrated on coaches using digital coaching, as practitioners are the most valuable resource for defining a term (Jones et al., 2019). However, asking the wider coaching ecosystem, such as coaches not using digital coaching, clients (not) using digital coaching, companies (not) offering digital coaching to their employees, and developers of digital coaching apps/platforms/offers can be of interest as well. A second limitation concerns the methodology of using a qualitative inductive approach with open-ended questions. Although this is an essential approach when defining a new topic, it does not allow comparability and quantification (Krippendorff, 2018). A third limitation addresses the method of asking in a survey instead of in an interview. Whilst in a survey, the situation and answers are more standardized, less biased, and easier to code, an in-person interview would have the opportunity to ask follow-up questions (Hyman and Sierra, 2016).

## Future research

Defining and differentiating digital coaching is only the first step for digital coaching research. For one thing, future research needs to explore coaching effectiveness among digital coaching compared to face-to-face coaching, hybrid forms, AI coaching, and other digital development interventions. For instance, it is yet unclear why face-to-face coaching and digital coaching are perceived as the same process and, yet, face-to-face sessions are significantly longer on average compared to digital coaching sessions based on our results. When exploring digital coaching effectiveness, aspects concerning the UN sustainable development goals (General Assembly Economic and Social Council, 2023) such as sustainability and inclusion can be investigated based on the advantages of digital coaching in terms of flexibility and accessibility.

Secondly, research is needed to compare the different digital coaching methods, as coaches use different digital technology tools in their digital coaching. This diversity is reflected in the different descriptions provided in the data: While most coaches point out the visual communication, others use digital coaching in the form of phone or audio coaching; additionally, while some use add-on support or even AI support, others do not. These variations in how digital coaching is used underscore the need for further research to identify and assess the advantages and disadvantages of these respective coaching approaches. Conducting interviews with experienced coaches could yield insights into their individual perspectives and experiences. These interviews could help identify common features and significant aspects of digital coaching. This could further contribute to a more differentiated definition. Furthermore, such interviews could also unveil emerging trends and developments in the realm of digital coaching, offering valuable insights for clarification and advancement within this field.

Thirdly, digital coaching process research is needed to investigate the best time to use digital sessions versus face-to-face sessions. For instance, it might or might not be essential to have a first session face-to-face in order to build up the trust and the relationship, which seems to be a hurdle in the digital coaching process based on our qualitative responses.

Fourthly, future research is needed to identify coach competencies that may be additionally needed (e.g., technological competencies) or need to be enhanced (e.g., empathic accuracy) when coaching digitally. For example, the qualitative responses of this study addressed a skill-set needed for digital coaching and coaching federations have started programs for their coaches in this regard (e.g., COACH-IT program; Muehl, n.d.).

Fifthly, as the realm of digital coaching continues to expand, ethics in digital coaching need to be considered. Integrating ethical principles ensures that users are treated with respect, privacy is safeguarded, and interventions are tailored to their needs. An example of a situation that should be clearly discussed with the client beforehand is recording the session, which can be very beneficial (van Coller-Peter and Manzini, 2020). In this case, obtaining prior consent and transparently clarifying how the recording will be used, as well as how it will be deleted, is advisable (Iordanou et al., 2017). Coaches need to maintain constant awareness regarding the potential risks to confidentiality in the virtual space where coaching takes place (Hawley et al., 2023). The potential for manipulation and data misuse underscores the necessity of a robust ethical framework.

### Practical implications

A clear definition of digital coaching can have several practical implications in the field. Firstly, it can provide a framework for coaches to understand their role and responsibilities in the digital coaching context (e.g., COACH-IT program; Muehl, n.d.). Such a framework can for example help coaches meet the specific needs of their clients and assist them in achieving their goals (e.g., GROW model; Whitmore, 2017). Secondly, a definition of digital coaching can guide the development of coaching protocols for digital interventions, which can ensure that coaches are properly trained, communication with clients is structured effectively, and adherence to guidelines is monitored (Lattie et al., 2019). Thirdly, a definition of digital coaching can help the clients’ understanding of what to expect from a digital coaching, supporting clients to make informed decisions about whether digital coaching, face-to-face coaching, or even AI coaching is a better fit. This understanding is essential for clearing expectations, as some expectations might only be met face-to-face and not in a digital environment (Murphy et al., 2021). Fourthly, a precise definition of digital coaching can assist in legal and regulatory matters. It might help in determining the boundaries and responsibilities of digital coaches, protecting both practitioners and clients. Fifthly, a definition of digital coaching can inform the design and development of digital coaching systems by identifying important functionalities, such as exercise programs and goal setting. Overall, a clear definition of digital coaching can enhance the effectiveness and quality of coaching practices in the digital realm.

## Data availability statement

The raw data supporting the conclusions of this article is available with the CC-BY Creative Commons under osf.io/nkj9a.

## Ethics statement

Ethical approval was not required for the studies involving humans because we only asked about a definition online. The studies were conducted in accordance with the local legislation and institutional requirements. The participants provided their written informed consent to participate in this study.

## Author contributions

JP developed the study’s conception and research idea, as well as provided the funding of the study (intercoder funding and open access funding). Both authors decided on the study’s design. SD took care of the material preparation, data collection, data analysis (supervision of the intercoders & quantitative analysis), the first draft of the manuscript, and the review process. All authors commented on versions of the manuscript as well as read and approved the final manuscript.

## Footnotes

^1^We had to exclude one answer for these two questions that was over 4.000 for both questions, suggesting that this person might have misinterpreted the question.

^2^ www.qcamap.org

^3^ osf.io/nkj9a
